# *Akkermansia muciniphila* promotes type H vessel formation and bone fracture healing by reducing gut permeability and inflammation

**DOI:** 10.1242/dmm.043620

**Published:** 2020-12-01

**Authors:** Jiang-Hua Liu, Tao Yue, Zhong-Wei Luo, Jia Cao, Zi-Qi Yan, Ling Jin, Teng-Fei Wan, Ci-Jun Shuai, Zheng-Guang Wang, Yong Zhou, Ran Xu, Hui Xie

**Affiliations:** 1 Department of Orthopedics, Xiangya Hospital, Central South University, Changsha, Hunan, 410008, China; 2 Movement System Injury and Repair Research Center, Xiangya Hospital, Central South University, Changsha, Hunan 410008, China; 3 Department of Stomatology, Xiangya Hospital, Central South University, Changsha, Hunan 410008, China; 4 State Key Laboratory of High Performance Complex Manufacturing, College of Mechanical and Electrical Engineering, Central South University, Changsha, Hunan 410008, China; 5 Department of Orthopedics, The Third Xiangya Hospital, Central South University, Changsha, Hunan 410013, China; 6 Department of Urology, The Second Xiangya Hospital, Central South University, Changsha, Hunan 410008, China; 7 Department of Sports Medicine, Xiangya Hospital, Central South University, Changsha, Hunan 410008, China; 8 Hunan Key Laboratory of Organ Injury, Aging and Regenerative Medicine, Changsha, Hunan 410008 China; 9 Hunan Key Laboratory of Bone Joint Degeneration and Injury, Changsha, Hunan 410008, China; 10 National Clinical Research Center for Geriatric Disorders, Xiangya Hospital, Central South University, Changsha, Hunan 410008, China

**Keywords:** *Akkermansia muciniphila*, Fracture healing, Type H vessel, Gut permeability, Inflammation

## Abstract

Improving revascularization is one of the major measures in fracture treatment. Moderate local inflammation triggers angiogenesis, whereas systemic inflammation hampers angiogenesis. Previous studies showed that *Akkermansia muciniphila*, a gut probiotic, ameliorates systemic inflammation by tightening the intestinal barrier. In this study, fractured mice intragastrically administrated with *A. muciniphila* were found to display better fracture healing than mice treated with vehicle. Notably, more preosteclasts positive for platelet-derived growth factor-BB (PDGF-BB) were induced by *A. muciniphila* at 2 weeks post fracture, coinciding with increased formation of type H vessels, a specific vessel subtype that couples angiogenesis and osteogenesis, and can be stimulated by PDGF-BB. Moreover, *A. muciniphila* treatment significantly reduced gut permeability and inflammation at the early stage. Dextran sulfate sodium (DSS) was used to disrupt the gut barrier to determine its role in fracture healing and whether *A. muciniphila* still can stimulate bone fracture healing. As expected, *A. muciniphila* evidently improved gut barrier, reduced inflammation and restored the impaired bone healing and angiogenesis in DSS-treated mice. Our results suggest that *A. muciniphila* reduces intestinal permeability and alleviates inflammation, which probably induces more PDGF-BB^+^ preosteoclasts and type H vessel formation in callus, thereby promoting fracture healing. This study provides the evidence for the involvement of type H vessels in fracture healing and suggests the potential of *A. muciniphila* as a promising strategy for bone healing.

This article has an associated First Person interview with the first author of the paper.

## Abstract (precis)

**Summary:** *Akkermansia muciniphila* reduces intestinal permeability and alleviates inflammation, inducing more PDGF-BB^+^ preosteoclasts and type H vessel formation in callus, thus promoting bone fracture healing.

## INTRODUCTION

With the rapid development of industry and transportation, the amount of bone fractures has remarkably increased in recent decades (Zhang et al., 2016). Although treatment techniques and implantation materials have been constantly improved (Gu et al., 2012; Hayes and Richards, 2010; Wing-Hoi et al., 2011; Zhang et al., 2016), some patients with fractures still exhibit delayed healing or nonunion (Wang et al., 2016; Yuasa et al., 2015), which causes great medical and economic cost (Bhandari et al., 2017). Therefore, the promotion of fracture healing has become a worldwide challenge with great clinical and social significance.

An adequate blood supply is supposed to be a mediator for optimal fracture repair (Lim et al., 2017; Tomlinson et al., 2013). Owing to the injury of vessels and vasoconstriction, blood flow evidently diminishes in the first few days post fracture (Claes et al., 2012). Approximately 2 weeks post fracture (WPF), the blood supply of the fracture site peaks and then gradually decreases to a normal level (Claes et al., 2012; Lim et al., 2017). At the early stages, a certain degree of inflammatory response is required to provoke an angiogenesis cascade (Cottrell and O'Connor, 2010), whereas systemic or additional local inflammation can inhibit revascularization and impair fracture healing (Lim et al., 2017; Loi et al., 2016). Normally, damage to vessels and other soft tissue surrounding the fracture zone induces local inflammation, which is considered to be an initial cause of new blood vessel formation. However, previous studies showed that severe fracture or multiple traumatic fractures can increase intestinal permeability, causing bacterial translocation and systemic inflammation (Levy et al., 2006; Napolitano et al., 1996; Wrba et al., 2018). Restoring the gut barrier has been demonstrated to improve several physical functions and ameliorate pathological changes, such as extending life span, sensitizing cancer to immune therapy and attenuating non-alcoholic steatohepatitis (Akagi et al., 2018; Bhutiani et al., 2018; Peng et al., 2018). Thus, we hypothesized that fracture-induced systemic inflammation, at least to some extent, may hamper bone repair. We also supposed that tightening intestinal barrier integrity may promote fracture healing by reducing inflammatory responses.

Previous studies showed that a specific vessel subtype named type H, which highly expresses CD31 and Endomucin (Emcn), couples angiogenesis and osteogenesis (Kusumbe et al., 2014; Ramasamy et al., 2014). We previously proved that the preosteoclasts (POC), tartrate-resistant acid phosphatase^+^ (TRAP^+^) mononuclear cells, release platelet-derived growth factor-BB (PDGF-BB) to induce formation of this capillary subtype (Xie et al., 2014). Moreover, we and others revealed that pharmacological measures to increase type H vessels can obviously enhance the bone formation in both aged and ovariectomized mice (Huang et al., 2018; Kusumbe et al., 2014; Xie et al., 2014). Improved fracture healing also requires robust vessel formation (Claes et al., 2012). However, the evidence for the involvement of type H vessels during the fracture healing process is still lacking.

In recent years, researchers have reached a consensus that the gut microbiota (GM) exerts a profound effect on the health status of the host (Gilbert et al., 2018). In terms of the skeletal system, a previous study showed that a low dose of antibiotic treatment in mice at weaning shifts the composition of the GM and causes high bone mass (Cho et al., 2012). Supplementation with probiotics prevents sex steroid depletion-induced bone loss via maintaining the gut barrier, inhibiting inflammation and suppressing osteoclastogenesis (Britton et al., 2014; Li et al., 2016b). *A*. *muciniphila* is a gram-negative anaerobic commensal, accounting for 1% to 5% of human GM (Derrien, 2004; Plovier et al., 2017). It has been proved that this mucin-degrading microbe can thicken the mucosal layer and tighten the gut barrier (Derrien et al., 2017). Recently, the importance of its role in the intestinal ecosystem has been highly appreciated. Multiple studies have indicated that *A. muciniphila* has a wide range of biological activities, including ameliorating atherosclerotic lesions by attenuating lipopolysaccharide (LPS)-induced inflammation, preventing alcoholic liver disease through enhancing mucus thickness and tightening the gut barrier, as well as improving metabolism in obese and diabetic mice (Grander et al., 2018; Li et al., 2016a; Plovier et al., 2017). Although an accumulation of evidence has illustrated the impacts of GM on bone and the benefits of *A. muciniphila* to health, the effect of *A. muciniphila* on bone fracture healing has not been reported. The effect of *A. muciniphila* on the intestinal epithelial barrier provides motivation for studying whether it can mitigate fracture operation-induced increasing gut permeability and systemic inflammation, and eventually strengthen bone repair.

In this study, we assessed whether *A. muciniphila* administration can promote fracture healing. We also tested the impact of this probiotic on PDGF-BB-releasing preosteoclasts and type H vessels. To identify the possible reasons of pro-osteogenic and pro-angiogenic effects of *A. muciniphila*, the inflammatory responses and gut permeability were detected. Additionally, the impact of another probiotic on fracture healing was tested to investigate whether the fracture healing-promoting effect is specific to *A. muciniphila.* We also used dextran sulfate sodium (DSS) to disrupt the gut barrier to determine whether *A. muciniphila* is still effective in promoting fracture healing. This study aimed to determine the therapeutic role of *A. muciniphila* in bone repair and to preliminarily explore the underlying mechanism.

## RESULTS

### Mice treated with *A. muciniphila* display improved quality of fracture healing

**Fig. 1.**
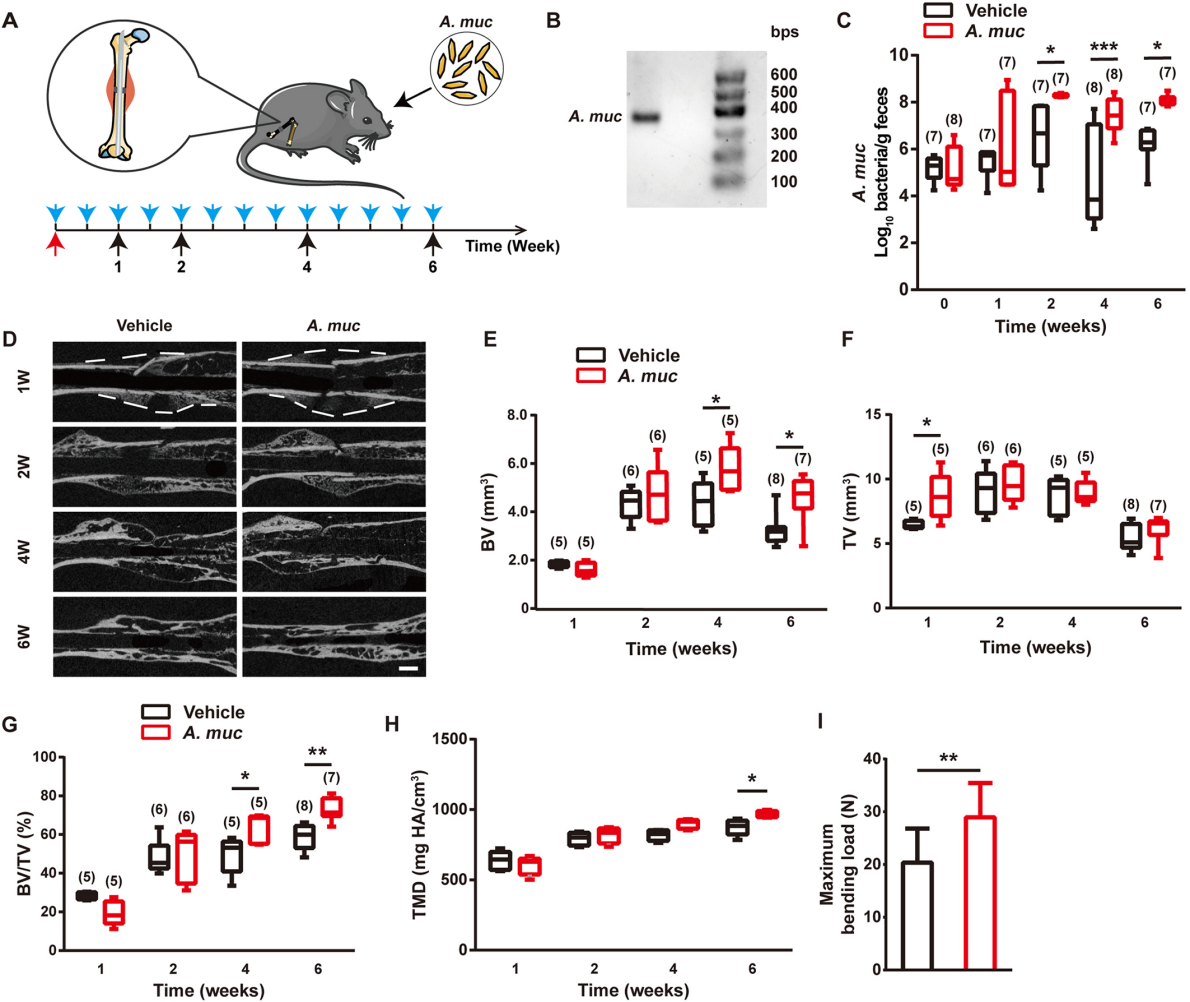
***A. muciniphila* treatment accelerates fracture healing*.*** (A) Experiment design for testing the impact of *A. muciniphila* on fracture healing. The blue arrows indicate *A. muciniphila* administrations. The red arrow indicates the fracture surgery time point. The black arrows indicate time points to sacrifice mice. (B) Amplifed *A. muciniphila* (*A. muc*) DNA band detected by agarose gel electrophoresis. (C) Abundance of *A. muciniphila* in fecal samples was examined by qRT-PCR. The *n* of each group is shown in parentheses. (D) Representative µCT images of fractured femora from vehicle- or *A. muciniphila*-treated mice at different time points. Scale bar: 1 mm. (E-G) µCT quantitative analyses of BV (E), TV (F) and BV/TV (G) of fractured femora at 1, 2, 4 and 6 WPF. The *n* of each group is shown in parentheses. (H) Tissue mineral density (TMD) of callus at 1, 2, 4 and 6 WPF. *n*=5 per group. (I) Four-point bending measurement of femoral ultimate load at 6 WPF. *n*=10 per group. Data are mean±s.d. Whiskers represent minimum and maximum values, and boxes represent the interquartile range. *P<0.05, **P<0.01, ***P<0.001, two-way ANOVA with Bonferroni post-hoc test (C,E-H), or unpaired two-tailed Student's *t*-test (I).

To study the impact of *A. muciniphila* on fracture healing, we challenged specific-pathogen-free (SPF) 8-week-old female mice with osteotomy surgery and intramedullary implantation. All fractured mice were treated with either *A. muciniphila* or the same volume of vehicle twice a week. Fecal samples were collected at four time points (1, 2, 4 and 6 WPF) and then all mice were sacrificed (Fig. 1A). For characterization of *A. muciniphila*, we extracted bacterial DNA from cultured *A. muciniphila* for PCR amplification. The PCR product was subjected to agarose gel electrophoresis with a DNA marker ranging from 100 to 600 bps. The resulting gel showed a DNA band between 300 bps and 400 bps, which is consistent with the fractional volume (329 bps) between the forward and reverse primers in the genomic DNA of *A. muciniphila* (Fig. 1B). Quantitative RT-PCR (qRT-PCR) analyses indicated that the *A. muciniphila* abundance in faeces was significantly elevated by *A. muciniphila* supplementation at 2, 4 and 6 WPF (Fig. 1C). These data indicated that *A. muciniphila* had successfully colonized the gut of mice.

To compare fracture healing quality between the *A. muciniphila* group and vehicle group, µCT analyses were performed to measure mineralized bone volume in callus (BV), total callus volume (TV) and BV/TV of callus (Zhang et al., 2016). At 1 WPF, µCT results showed greater TV in the *A. muciniphila* group than in the vehicle group, but no significant difference of BV was found between the two groups (Fig. 1D-F). At 4 and 6 WPF, the BV of the *A. muciniphila* group was much higher than the vehicle group (Fig. 1E). The TV of both the *A. muciniphila-* and vehicle-treated mice peaked at 2 WPF, and then gradually decreased, probably owing to callus remodeling (Fig. 1F). No statistical difference of TV was observed between the two groups from 2 WPF (Fig. 1F). The value of BV/TV in the *A. muciniphila* group was slightly lower at 1 WPF but significantly greater at 4 and 6 WPF than the vehicle group (Fig. 1G), which was attributed to the promoted BV of the *A. muciniphila* group at later stages. Tissue mineral density analysis shows that *A. muciniphila* treatment enhanced callus mineralization at 6 WPF (Fig. 1H). Next, we performed a four-point bending test at 6 WPF to test the biomechanical properties of fractured femora. As shown in Fig. 1I, the maximum bending load of fractured femora in the *A. muciniphila* group was significantly greater than in the vehicle group. These results demonstrated that *A. muciniphila* supplementation of a mouse femur fracture model resulted in more callus formation at an early stage, and accelerated mineralization at later stages, as compared to vehicle treatment. We also performed 16S rDNA qRT-PCR analysis with a series of phylum- and class-specific primers (Bacchetti De Gregoris et al., 2011) to determine whether the *A. muciniphila* supplementation altered the gut microbiome. As shown in Fig. S1A, *A. muciniphila* induced a marked increase of Actinobacteria, which was consistent with a previous study (Wu et al., 2020). In addition, *A. muciniphila* colonization reduced the abundance of γ-Proteobacteria and led to an increasing trend of Bacteroidetes.

To confirm the direct effect *A. muciniphila* exerted on fracture healing, an antibiotic (ABX) cocktail (vancomycin, ampicillin, neomycin and metronidazole) was used to deplete the gut microbiota (Blacher et al., 2019). The mice were then fractured and the effect of *A. muciniphila* was re-tested. As shown in Fig. S1B-F, *A. muciniphila* still promoted bone fracture healing in ABX-treated mice. Both BV and TV were elevated at 6 WPF, resulting in an improved anti-bending property (Fig. S1B-F). qRT-PCR analyses showed that *A. muciniphila* successfully colonized the gut of ABX-treated mice (Fig. S1G). Our results suggest that *A. muciniphila* has the ability to stimulate fracture repair through an endogenous microbiota-independent mechanism.

### Mice treated with *A. muciniphila* display improved bone formation

**Fig. 2.**
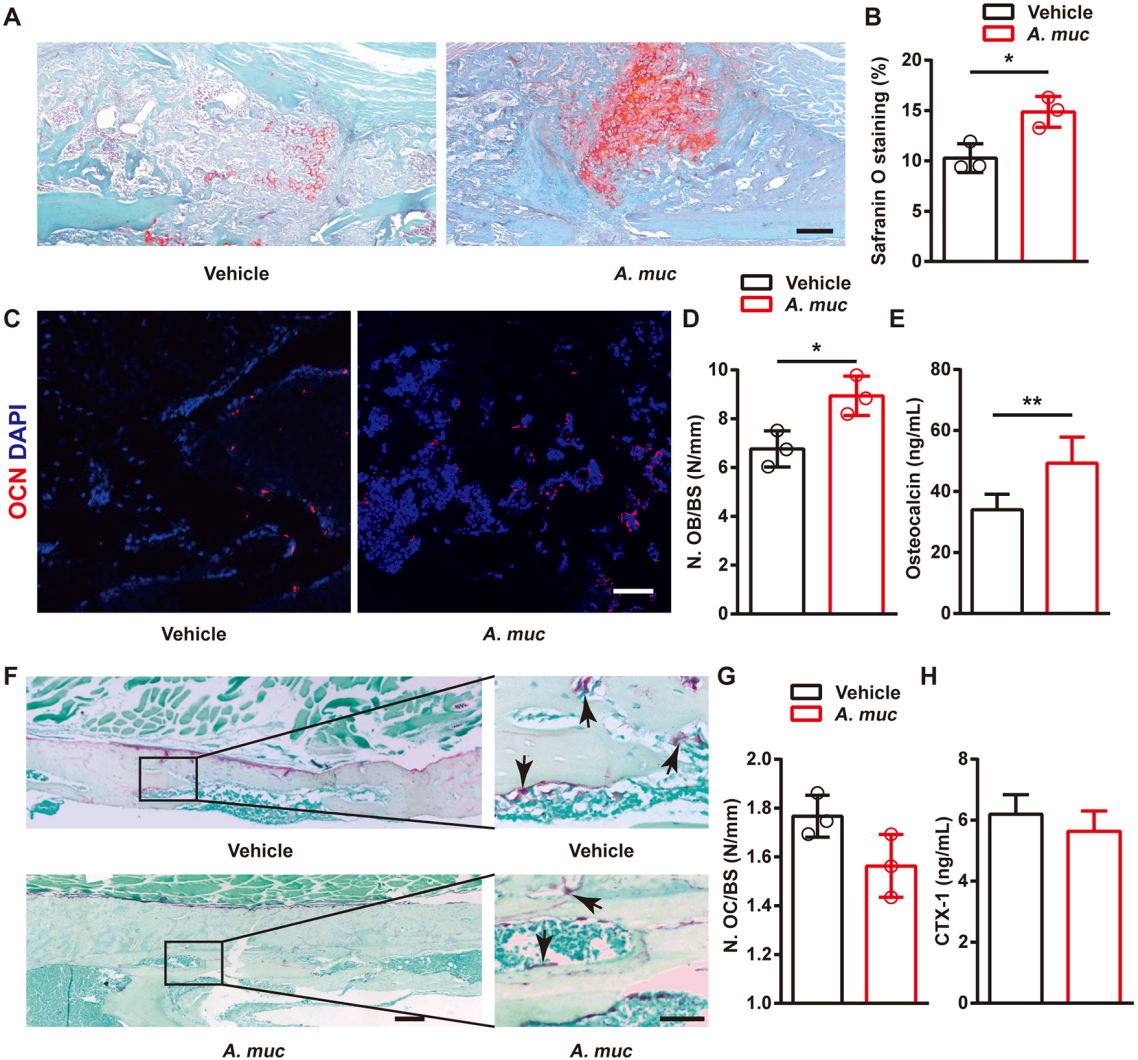
**The effects of *A. muciniphila* treatment on chondroid callus formation, osteogenesis and osteoclastogenesis in fractured femora of mice.** (A,B) Representative images of Safranin O staining (A) and quantification of the positive-stained area (B) in fractured femora from vehicle- or *A. muciniphila*-treated mice at 2 WPF. *n*=3 per group. (C,D) Representative OCN-stained sections (C) with quantification of osteoblast number (N. OB) (D) in fractured femora from vehicle- or *A. muciniphila*-treated mice at 6 WPF. *n*=3 per group. (E) Serum concentrations of OCN detected by ELISA at 6 WPF. *n*=6 per group. (F,G) Representative TRAP-stained sections (F) with quantification of osteoclast number (N. OC) (G) in fractured femora from vehicle- or *A. muciniphila*- treated mice at 6 WPF. Arrows indicate osteoclasts. *n*=3 per group. (H) Serum concentrations of CTX-1 detected by ELISA at 6 WPF. *n*=6 per group. Data are mean±s.d. *P<0.05, **P<0.01, unpaired two-tailed Student's *t*-test. Scale bars: 100 μm (A); 50 μm (C); 200 μm (left); 50 μm (right) (F).

We next investigated whether *A. muciniphila* treatment influenced chondroid callus formation, bone formation and bone resorption. Safranin O staining at 2 WPF showed more chondroid matrix was formed in fracture callus of *A. muciniphila*-treated mice compared to vehicle-treated mice (Fig. 2A,B), which is thought to benefit endochrondral ossification at later stages. Consistently, immunostaining of osteocalcin (OCN) showed more osteoblasts (the cells responsible for bone formation) in the callus area at 6 WPF in the *A. muciniphila* group compared with vehicle group (Fig. 2C,D). Enzyme-linked immunosorbent assay (ELISA) results showed that the serum level of OCN (serum marker of bone formation) was also significantly elevated by *A. muciniphila* treatment (Fig. 2E). TRAP staining indicated that the TRAP^+^ multinucleated cells (osteoclasts, the cells responsible for bone resorption) were not statistically significantly different between the *A*. *muciniphila* and vehicle groups at 6 WPF (Fig. 2F,G). Consistently, no significant difference in the C-terminal telopeptides of type 1 collagen in serum (CTX-1, serum marker of bone resorption) between the two groups could be detected (Fig. 2H). These data illustrated that supplementation of *A. muciniphila* improved fracture healing at both early and later stages, and better bone repair may be attributed to increased bone formation.

### *A. muciniphila* treatment stimulates type H vessel formation in callus

Type H vessels are responsible for supporting bone formation (Huang et al., 2018; Ramasamy et al., 2014; Xie et al., 2014). Our previous study showed that preosteoclasts could release PDGF-BB to induce formation of type H vessels for attenuating bone loss in sex steroid-deficient mice (Xie et al., 2014). However, the involvement of type H vessels in fracture repair has not been reported. Therefore, we investigated whether *A. muciniphila* treatment could stimulate type H vessel formation during fracture healing.

**Fig. 3.**
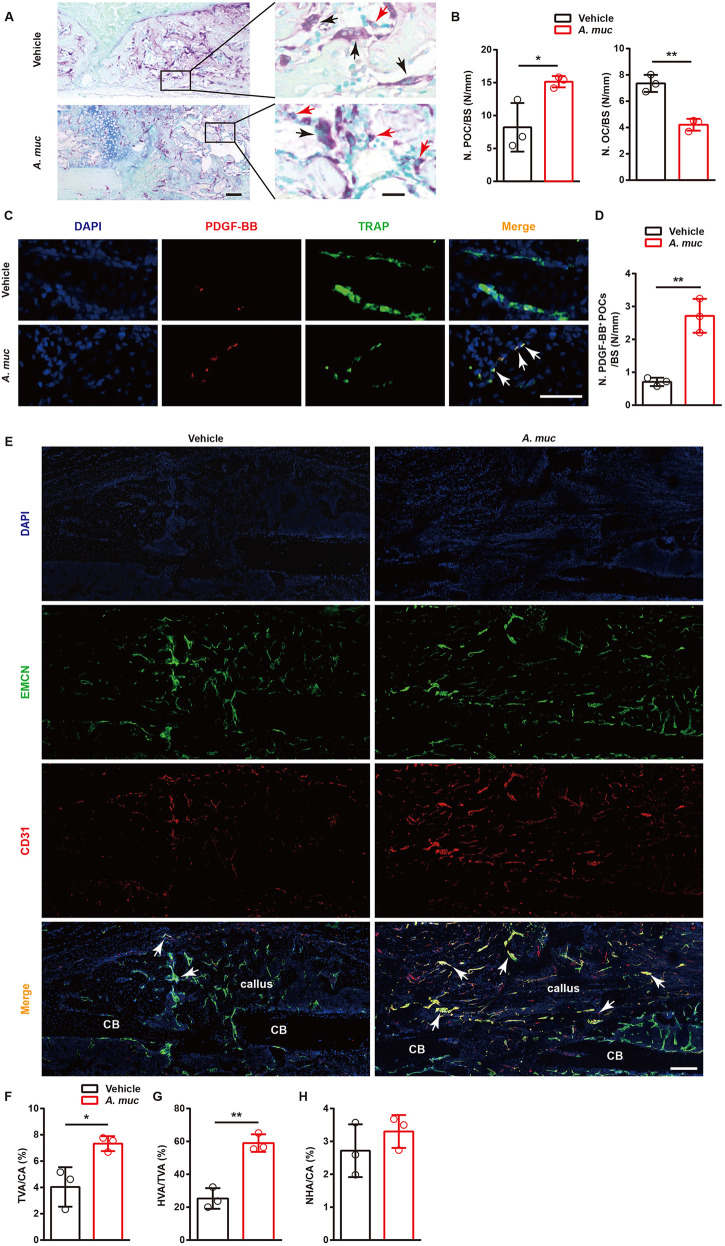
***A. muciniphila* supplementation stimulates preosteoclast and type H vessel formation in fractured femora of mice.** (A,B) Representative images of TRAP staining (A) with quantification of preosteoclast number (N. POC, left) and osteoclast number (N. OC, right) (B) at 2 WPF. The black arrows indicate osteoclasts and the red arrows indicate preosteoclasts. *n*=3 per group. (C,D) Representative images of TRAP^+^ (green) and PDGF-BB^+^ (red) co-immunostaining (C) with quantification of PDGF-BB^+^ preosteoclast (TRAP^+^ mononuclear cells) number (D) at 2 WPF. Arrows indicate PDGF-BB^+^ preosteoclast cells. *n*=3 per group. (E-H) Representative images of CD31 and Emcn co-immunostaining (E) with quantification of the ratio of total vessels area (TVA) (F), type H vessels area (HVA) (G) and non-type H vessels area (NHA) (H) at 2 WPF. Arrows indicate type H vessels. *n*=3 per group. Data are mean±s.d. *P<0.05, **P<0.01, unpaired two-tailed Student's *t*-test. BS, bone surface; CA, callus area; CB, cortical bone. Scale bars: 200 μm (left), 20 μm (right) (A); 50 μm (C); 200 μm (E).

As vessel formation in the fracture zone reaches a high level at ∼2 WPF (Lim et al., 2017), we verified the number of preosteoclasts and osteoclasts at this time point. TRAP staining showed that there were distinctly more preosteoclasts and less osteoclasts on the surface of woven bone in *A. muciniphila*-treated mice at 2 WPF compared to vehicle-treated mice (Fig. 3A,B). We previously reported that preosteoclasts stimulate type H vessel formation by secreting PDGF-BB. Therefore, co-immunostaining of TRAP and PDGF-BB at 2 WFP was conducted. As expected, more preosteoclasts (TRAP^+^ mononuclear cells) positive for PDGF-BB were detected on the surface of woven bone in the fracture area of *A. muciniphila*-treated mice than in vehicle-treated mice (Fig. 3C,D). Next, to determine the formation of type H vessels, co-immunostaining of CD31 (also known as Pecam1) and Emcn was performed at 2 WPF. The data showed that the formation of both total vessels and type H vessels were significantly higher in the fracture area of *A. muciniphila*-treated mice compared to vehicle-treated mice, whereas the formation of non-type H vessels was similar between the two groups (Fig. 3E-H). Taken together, *A. muciniphila* treatment effectively boosted type H vessel formation in callus, which was probably attributable to the increased number of PDGF-BB^+^ preosteoclasts in the fracture area.

### *A. muciniphila* supplemented mice exhibit a lower level of inflammatory responses at early stages of fracture healing

**Fig. 4.**
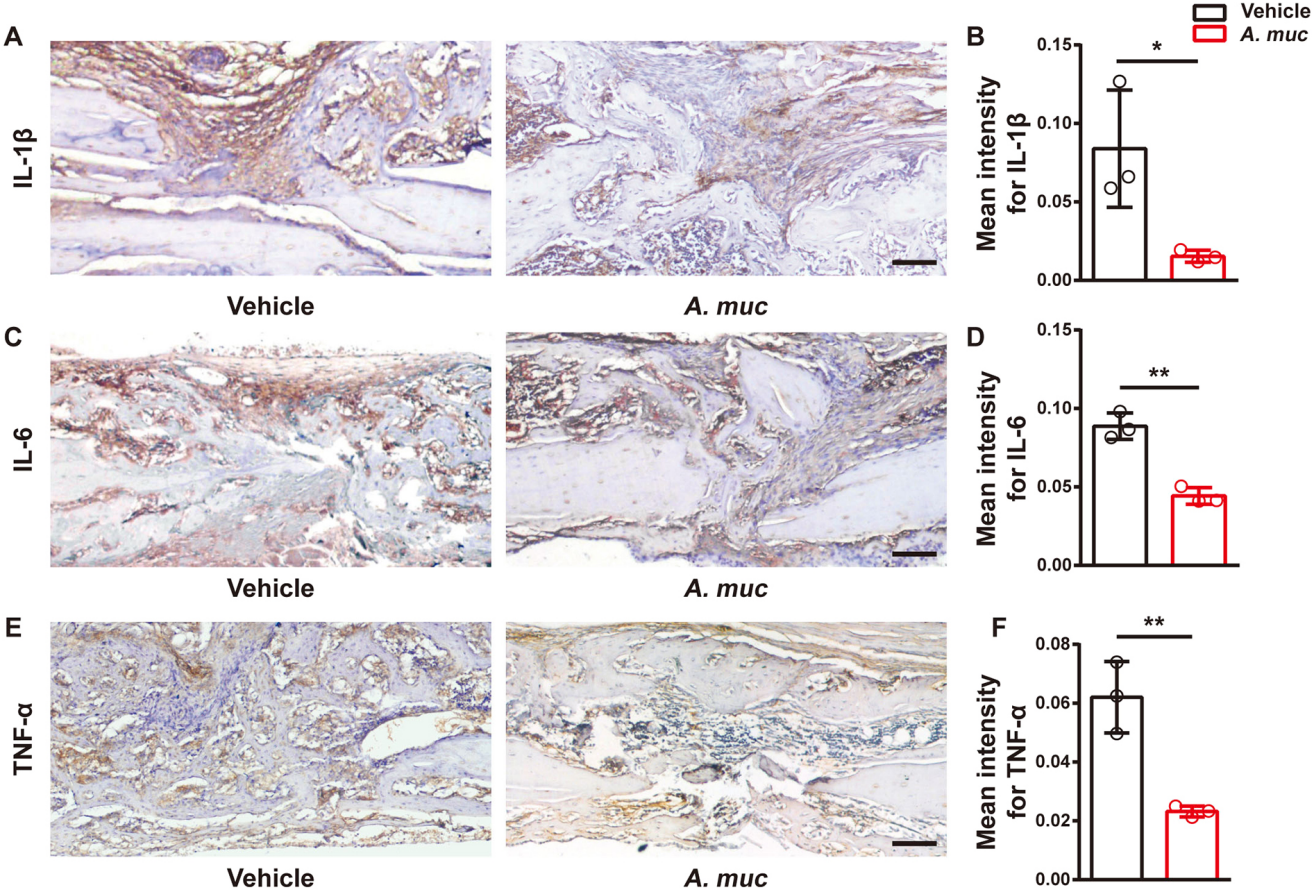
***A. muciniphila* treatment alleviates local inflammatory responses in fractured femora of mice.** (A,B) Representative images (A) and quantitative analysis (B) of immunohistochemical staining for IL1β at 2 WPF. *n*=3 per group. (C,D) Representative images (C) and quantitative analysis (D) of immunohistochemical staining for IL6 at 2 WPF. *n*=3 per group. (E,F) Representative images (E) and quantitative analysis (F) of immunohistochemical staining for TNF-α at 2 WPF. *n*=3 per group. Data are mean±s.d. *P<0.05, **P<0.01, unpaired two-tailed Student's *t*-test. Scale bars: 100 μm.

Excessive inflammation is considered to be one of the most important systemic factors that inhibit blood vessel formation during fracture healing (Claes et al., 2012; Schmidt-Bleek et al., 2015). Previous reports, including ours, have demonstrated that excessive inflammation stimulates preosteoclast fusion, thus promoting bone resorption (Li et al., 2016b; Rao et al., 2018). Given the anti-inflammatory effects of *A. muciniphila in vivo* (Grander et al., 2018; Li et al., 2016a), we measured the local inflammation level of the fracture site at 2 WPF. Immunohistochemical analyses showed weaker staining intensities of interleukin 1β (IL1β), IL6 and tumor necrosis factor-α (TNF-α) in the fracture area of the *A. muciniphila* group than in the vehicle group (Fig. 4A-F). Notably, all of these pro-inflammatory cytokines we tested have been previously shown to impair angiogenesis in fracture healing (Chambers et al., 2018; Kaiser et al., 2018; Lange et al., 2010; Lim et al., 2017; Prystaz et al., 2018).

Macrophage colony stimulating factor (M-CSF, also known as CSF1) is required by preosteoclasts for survival, proliferation and osteoclastic differentiation, whereas the receptor activator of nuclear factor-kappaB ligand (RANKL), another key osteoclastogenesis cytokine, is responsible for osteoclast formation and the activation of osteoclastic resorptive function (Kong et al., 2020). Therefore, we evaluated the level of M-CSF and RANKL in serum. The data shown in Fig. S2A,B demonstrate that both factors were significantly downregulated by *A. muciniphila* at 2 WPF, at which point *A. muciniphila* exerted an anti-inflammatory effect on the fracture site. Consistent with our results, multiple previous studies demonstrated that inflammation can stimulate production of M-CSF and RANKL (Meng et al., 2020; Mossadegh-Keller et al., 2013; Pietschmann et al., 2016). RANKL alone cannot induce osteoclastogenesis without M-CSF (Seo et al., 2007). These data, when taken together, suggest that the anti-inflammatory outcome of *A. muciniphila* treatment may be the reason for greater numbers of preosteoclasts and type H vessels, as well as accelerated fracture healing.

### *A. muciniphila* treatment decreases intestinal permeability and inflammation

**Fig. 5.**
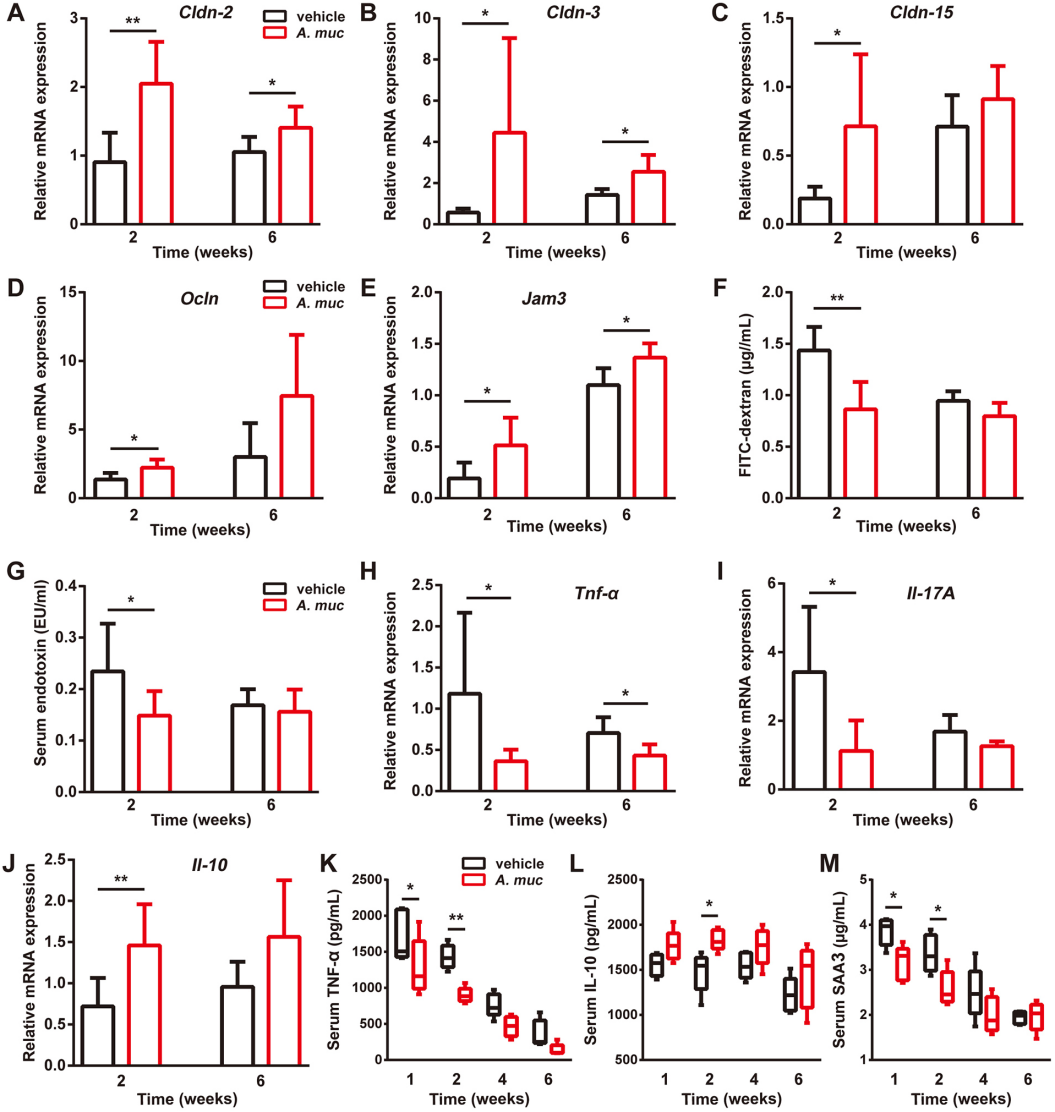
***A. muciniphila* administration tightens the gut barrier and reduces systemic inflammation in the femur fracture mouse model.** (A-E) qRT-PCR measurements of colonic mRNA levels of *Cldn-2*, *-3* and *-15*, as well as of *Ocln* and *Jam3* at 2 and 6 WPF. For 2 weeks, *n*=6 per group; for 6 weeks, *n*=7 per group. (F) Serum FITC-dextran levels were measured 4 h after FITC-dextran oral administration at 2 and 6 WPF. *n*=5 per group. (G) LPS levels in serum samples at 2 and 6 WPF. *n*=8 per group. (H-J) qRT-PCR analyses of colonic mRNA levels of *Tnf-α*, *Il17A* and *Il10* at 2 and 6 WPF. For 2 weeks, *n*=6 per group; for 6 weeks, *n*=7 per group. (K-M) Serum concentrations of TNF-α (K), IL10 (L) and SAA3 (M) detected by ELISA at 1, 2, 4 and 6 WPF. *n*=5 per group. Data are mean+s.d. Whiskers represent minimum and maximum values, and boxes represent the interquartile range. *P<0.05, **P<0.01, unpaired two-tailed Student's *t*-test (A-J) or two-way ANOVA with Bonferroni post-hoc test (K-M).

Previous studies showed that the anti-inflammatory effects of *A. muciniphila* are largely correlated to the restoration of the gut barrier function, rendering a correction of bacterial LPS under some pathological circumstances (Grander et al., 2018; Li et al., 2016a; Plovier et al., 2017). As fractures were proven to increase gut permeability, we analyzed the intestinal mRNA expression of a class of tight junction proteins, including occludin, jam3, claudin2, 3, and 15 (Li et al., 2016b; Plovier et al., 2017). The data shown in Fig. 5A-E demonstrate that intestinal mRNA levels of these factors were significantly higher in *A. muciniphila*-treated mice compared to vehicle-treated mice at 2 WPF. To further confirm the improved gut barrier induced by *A. muciniphila*, mice in these two groups were administrated orally with fluorescein isothiocyanate–dextran (FITC-dextran) at 2 and 6 WPF. After 4 h, serum concentrations of FITC-dextran were tested. As shown in Fig. 5F, vehicle-treated mice had a much higher serum level of FITC-dextran at 2 WPF, whereas the difference between the two groups at 6 WPF was not significant. A limulus amebocyte lysate (LAL) assay indicated that *A. muciniphila* treatment led to decreased circulatory LPS compared to vehicle treatment at 2 WPF but not at 6 WPF (Fig. 5G). Correspondingly, compared to vehicle-treated mice, *A. muciniphila*-treated mice exhibited significantly lower intestinal mRNA levels of pro-inflammatory factors TNF-α and IL17a, but significantly higher intestinal mRNA levels of anti-inflammatory factor IL10 at 2 WPF (Fig. 5H-J). The gut barrier tightening effect and intestinal anti-inflammatory effect of *A. muciniphila* at 6 WPF were not as strong as that at 2 WPF (Fig. 5A-J). To test the effect of *A. muciniphila* on systemic inflammation, serum samples were tested by ELISA. The results suggested that, at early stages, *A. muciniphila* elevates the level of anti-inflammatory factor IL10 and reduces the level of TNF-α and serum amyloid A3 (SAA3) (Fig. 5K-M), two factors thought to promote inflammation and stimulate osteoclastogenesis (Thaler et al., 2015; Thompson et al., 2018). Taken together, these data suggest that *A. muciniphila* treatment reduces gut permeability and systemic inflammation at early stages, at which point the angiogenesis of the fractured area reaches the highest level (Lim et al., 2017).

### Supplementation of probiotics promote bone fracture healing

**Fig. 6.**
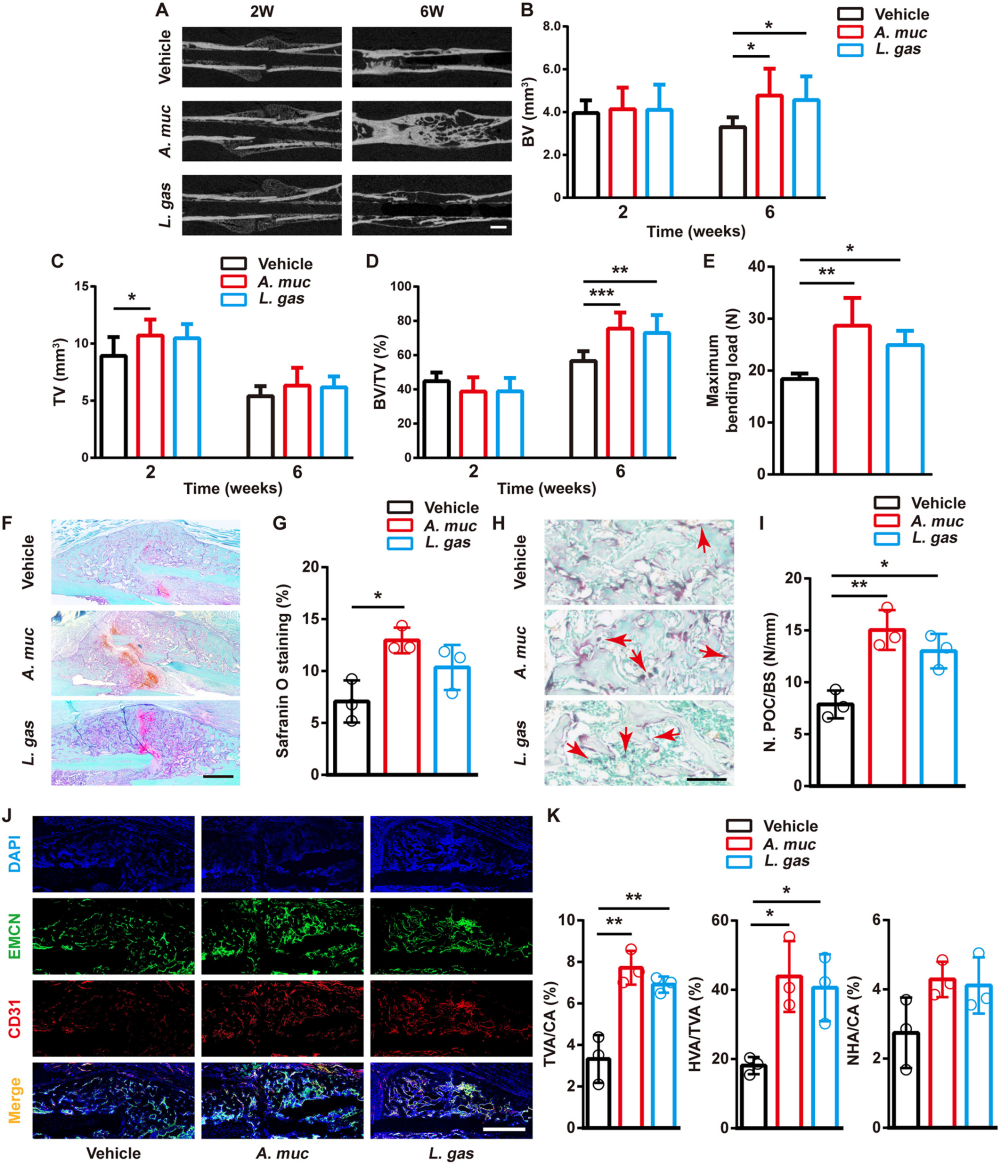
**Supplementation of probiotics promotes bone repair and angiogenesis.** (A) Representative µCT images of fractured femora from vehicle-, *A. muciniphila*- and *L. gasseri*-treated mice at 2 and 6 weeks. (B-D) µCT quantitative analyses of BV (B), TV (C) and BV/TV (D) of fractured femora at 2 and 6 WPF. *n*=8 per group. (E) Four-point bending measurement of femoral ultimate load at 6 WPF. *n*=5 per group. (F,G) Representative images of Safranin O staining (F) and quantification of the positive-stained area (G) in fractured femora at 2 WPF. *n*=3 per group. (H,I) Representative images of TRAP-staining (H) with quantification of preosteoclast number (N. POC) (I) at 2 WPF. Red arrows indicate preosteoclasts. BS, bone surface. *n*=3 per group. (J,K) Representative images of CD31 and Emcn co-immunostaining (J) with quantification (K) of the ratio of total vessels area (TVA), type H vessels area (HVA) and non-type H vessels area (NHA) at 2 WPF. *n*=3 per group. Data are mean±s.d. *P<0.05, **P<0.01, ***P<0.001, one-way ANOVA with Bonferroni post-hoc test. Scale bars: 1 mm (A); 200 μm (F); 50 μm (H); 500 μm (J).

We next investigated whether the improved fracture healing was specific to *A. muciniphila* and whether other probiotics that reduce gut permeability and inflammation could also promote bone repair. *Lactobacillus gasseri* was reported to ameliorate inflammation and obesity, tighten gut barrier integrity and improve glucose tolerance (Kawano et al., 2016; Shirouchi et al., 2016). Therefore, we re-evaluated the impact of *A. muciniphila* on fracture healing, with one additional control group of *L. gasseri* treatment. µCT scanning showed that mice that were treated with *L. gasseri* exhibited higher BV at 2 WPF and higher TV at 6 WPF, compared with vehicle-treated mice (Fig. 6A-D). Biomechanical testing also showed that the femora of *L. gasseri*-treated mice had better healing quality than that of vehicle-treated mice (Fig. 6E). Safranin O staining at 2 WPF showed that chondroid matrix formation in fracture callus was slightly increased by *L. gasseri* (Fig. 6F,G). We also found that *L. gasseri* treatment lead to more preosteoclasts in callus at 2 WPF (Fig. 6H,I). Similar to *A. muciniphila*, *L. gasseri* treatment promoted the formation of new vessels, especially type H vessels (Fig. 6J,K). These results suggest that *L. gasseri* treatment also has the ability to improve bone fracture healing.

### *A. muciniphila* treatment rescued gut barrier dysfunction-induced impairment of bone healing

**Fig. 7.**
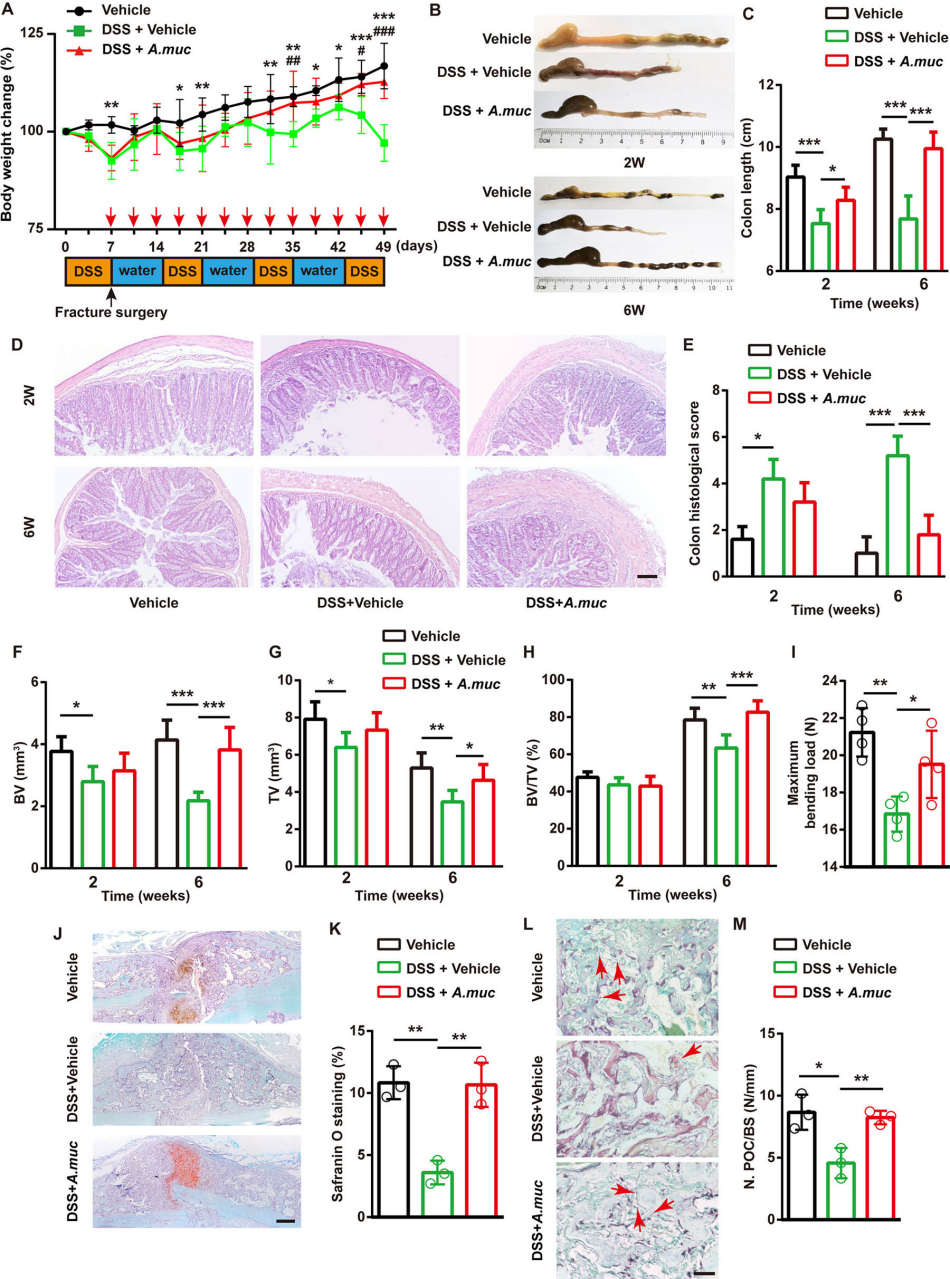
***A. muciniphila* protected against DSS-induced impairment on fracture healing.** (A) Experimental design for DSS treatment and body weight change of mice. Red arrows indicate *A. muciniphila* (*A. muc*) administration time points. Black arrow indicates fracture surgery time point. *n*=6 per group. (B,C) Representative images of colons (B) with quantification of colon length at 2 and 6 WPF (C). *n*=6 per group. (D,E) Representative images of HE staining of colon at 2 and 6 WPF (D) with quantification of histological scores (E). *n*=5 per group. (F-H) µCT quantitative analyses of BV (F), TV (G) and BV/TV (H) of fractured femora at 2 and 6 WPF. *n*=6 per group. (I) Four-point bending measurement of femoral ultimate load at 6 WPF. *n*=4 per group. (J,K) Representative images of Safranin O staining (J) and quantification of positive-stained area (K) in fractured femora at 2 WPF. *n*=3 per group. (L,M) Representative images of TRAP staining (L) with quantification of preosteoclast number (N. POC) (M) at 2 WPF. The red arrows indicate preosteoclasts. BS, bone surface. *n*=3 per group. *P<0.05, **P<0.01, ***P<0.001, DSS plus vehicle group versus vehicle group ^#^*P*<0.05, ^###^*P*<0.001, DSS plus vehicle group versus DSS plus *A. muciniphila* group. Data are mean+s.d. A two-way ANOVA with Bonferroni post-hoc test was used to perform multiple-group comparisons (A) and an unpaired two-tailed Student's *t*-test was used in C, E, F-I and K. Scale bars: 100 μm (D,J); 20 μm (L).

To understand the role of the gut barrier in fracture repair and whether *A. muciniphila* is still effective when intestinal permeability increases, DSS was employed to disrupt the intestinal barrier. All the mice were divided into three groups: vehicle group, DSS plus vehicle group and DSS plus *A. muciniphila* group. Before surgery and *A. muciniphila* administration, a 7-day DSS treatment (2.5% w/v) was given to the DSS plus vehicle and DSS plus *A. muciniphila* groups. After surgery, three rounds of DSS treatment (8-day normal drinking water followed by 6-day DSS) were performed. Mice in the vehicle group only drank normal water. As shown in Fig. 7A, all the mice that received DSS exhibited a significantly lower body weight at early stages. However, by day 35, mice in the DSS plus *A. muciniphila* group began to exhibit a higher body weight than the DSS plus vehicle group. At late stages of the experiment, mice in the DSS plus *A. muciniphila* group had a similar body weight to the vehicle group (Fig. 7A). DSS treatment significantly shortened the colon, whereas *A. muciniphila* treatment significantly alleviated intestinal damage (Fig. 7B,C). Hematoxylin eosin (HE) staining of colonic sections showed that *A. muciniphila* treatment distinctly rescued the mucosal damage and intestinal inflammation induced by DSS, at both 2 and 6 WPF (Fig. 7D,E).

µCT results show that DSS hampered bone healing, as indicated by less BV and TV at both 2 and 6 WPF (Fig. 7F,G). The DSS plus *A. muciniphila* group mice exhibited much higher BV and TV than the DSS plus vehicle group mice, and even mildly higher BV/TV than vehicle group mice at 6 WPF (Fig. 7F-H). A four-point bending test suggested that *A. muciniphila* significantly enhanced biomechanical properties in DSS-treated mice (Fig. 7I). Additionally, *A. muciniphila* treatment restored chondroid matrix formation and the number of preosteoclasts, as evidenced by Safranin O and TRAP staining (Fig. 7J-M).

**Fig. 8.**
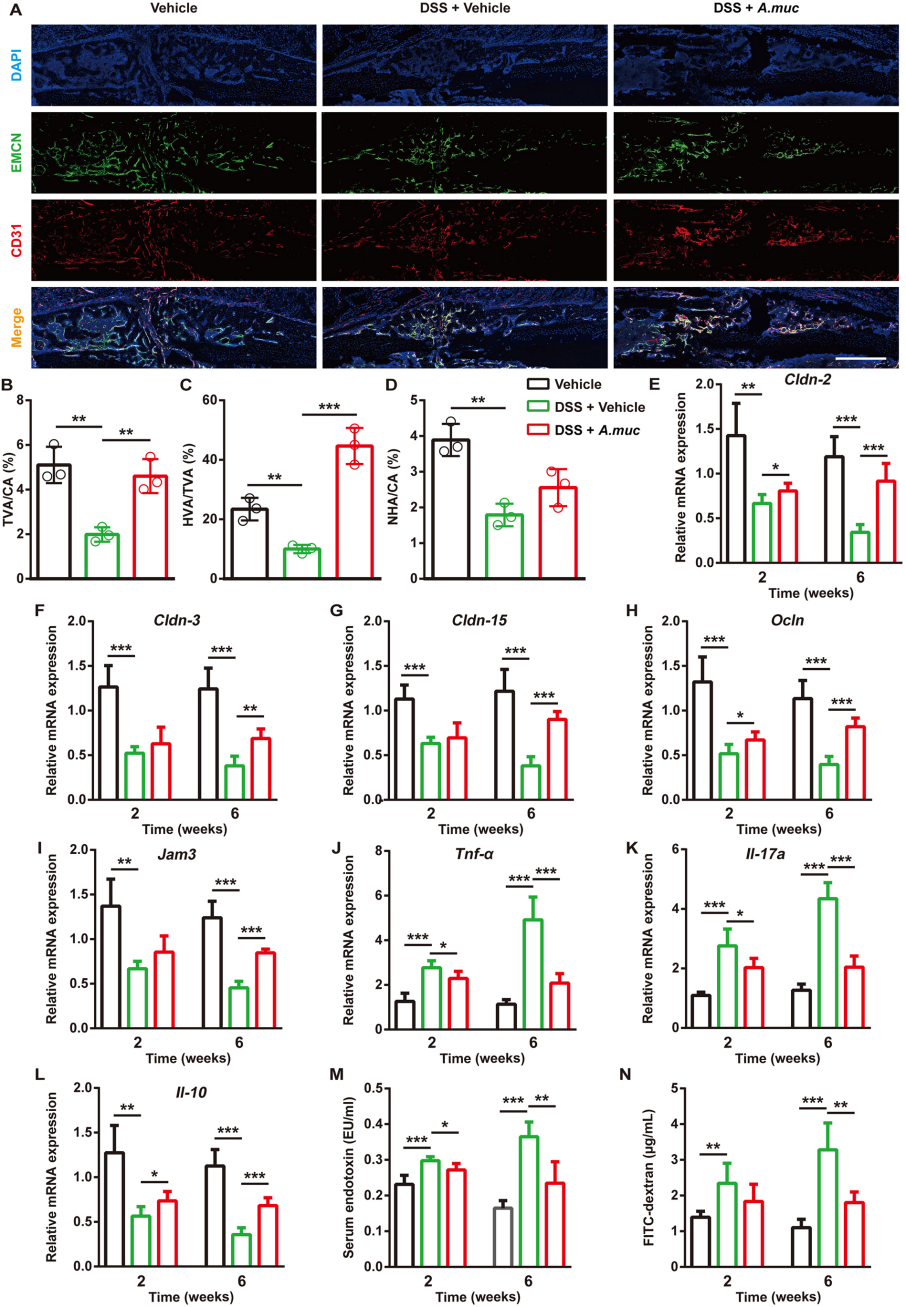
***A. muciniphila* treatment restores the type H vessels formation and decreases the gut permeability of DSS-treated mice.** (A-D) Representative images of CD31 and Emcn co-immunostaining (A) with quantification of the ratio of total vessels area (TVA), type H vessels area (HVA) and non-type H vessels area (NHA) (L) at 2 WPF. *n*=3 per group. Scale bar: 500 μm. (E-L) qRT-PCR measurements of colonic mRNA levels of *Cldn-2*, *-3* and *-15*, *Ocln*, *Jam3*, *Tnf-α*, *Il17A* and *Il10* at 2 and 6 WPF. *n*=5 per group. (M) LPS levels in serum samples at 2 and 6 WPF. *n*=5 per group. (N) Serum FITC-dextran levels were measured 4 h after FITC-dextran oral administration at 2 and 6 WPF. *n*=5 per group. Data are mean±s.d. *P<0.05, **P<0.01, ***P<0.001, unpaired two-tailed Student's *t*-test.

We also tested the angiogenesis of the three groups. As shown in Fig. 8A-D, DSS treatment significantly reduced new vessel formation, including the type H and non-type H vessels. *A. muciniphila* treatment attenuated the loss of angiogenesis caused by DSS, largely due to type H vessels (Fig. 8A-D). Next, a class of gut barrier and inflammation-related mRNA was tested. The data demonstrated that *A. muciniphila* treatment tightened impaired gut barrier integrity and decreased colonic inflammation in DSS-treated mice (Fig. 8E-L). Consistently, the serum LPS and serum FITC-dextran levels also indicated that *A. muciniphila* treatment reduced intestinal permeability (Fig. 8M,N). This body of evidence suggests that *A. muciniphila* treatment can promote bone repair and type H vessel formation via tightening the gut barrier and decreasing inflammation.

## DISCUSSION

Recent advances in understanding how gut microbiota contribute to the physiological and pathological state of the host have generated great attention for the employment of probiotics to improve human health. Probiotics have been identified as ‘live microorganisms, which upon ingestion in certain numbers, exert health benefits beyond inherent basic nutrition’ (Guarner and Schaafsma, 1998). Several probiotics have shown their bone-sparing effects, such as anti-osteoporosis and enhancing bone mass (Li et al., 2016b; Ohlsson et al., 2014). However, the effect of probiotics on fracture healing is less well studied. Over the past few years, *A. muciniphila* has been introduced as a next-generation probiotic because of its positive bioactivities on host health (Naito et al., 2018), especially the anti-inflammatory effect. Previous studies have demonstrated that supplementation with *A. muciniphila* can ameliorate several inflammation-related diseases by enhancing colonic mucus thickness and tightening the gut barrier (Grander et al., 2018; Li et al., 2016a; van der Lugt et al., 2019). In this study, we showed that *A. muciniphila* supplementation improves bone fracture healing in both normal SPF mice and gut microbiota-depleted mice, and this provides a new approach for bone-related disease treatment and broadens the potential clinical application of *A. muciniphila*.

The process of fracture repair consists of three partially overlapping periods: inflammation, repair and remodeling (Claes et al., 2012). Once fracture occurs, fracture hematoma is gradually formed, caused by bone-surrounding soft tissue injury and hemorrhage (Paula et al., 2010; Richard and Einhorn, 2011). Injuries-initiated inflammation is thought to be positive for angiogenesis and fracture repair when it is moderate (Loi et al., 2016). However, previous studies reported that bone fractures weaken gut barrier integrity, leading to bacterial translocation-caused systemic inflammation (Levy et al., 2006; Napolitano et al., 1996; Wrba et al., 2018). In this study, we firstly applied *A. muciniphila* to treat fractured mice, and found that fractured mice supplemented with *A. muciniphila* display an improved gut epithelial barrier integrity and reduced inflammatory responses. Previous studies showed that *A. muciniphila* and *A. muciniphila*-derived extracellular vesicles can alleviate DSS-induced colitis (Chelakkot et al., 2018; Kang et al., 2013; Zhai et al., 2019), which is characterized by mucosal damage and inflammation infiltration. In our study, *A. muciniphila* significantly preserved the gut barrier and restored the loss of endochondral ossification and angiogenesis induced by DSS. Consistently, *A. muciniphila* treatment caused less osteoclasts in the fracture area at 2 WPF, in agreement with previous results that showed osteoclast generation is augmented in inflammatory conditions, and inhibiting inflammation can prevent osteoclastogenesis (Li et al., 2016b; Rao et al., 2018). Our data suggest that a tightened gut barrier is of great importance for fracture healing, which provides a potential explanation and therapeutic tool for delayed union and non-union. We also found *L. gasseri*, another probiotic that has also been shown to reduce intestinal permeability and inflammation, exerts a pro-angiogenesis effect and promotes bone healing, implying that not only *A. muciniphila*, but also other probiotics can benefit bone repair. Notably, *L. gasseri* shows a trend towards a decreased fracture healing-promoting effect compared to *A. muciniphila*.

The importance of angiogenesis in fracture healing has long been highly appreciated. Previous studies demonstrated that the type H vessel couples angiogenesis and osteogenesis, and mediates the local growth of vasculature (Kusumbe et al., 2014; Ramasamy et al., 2014). We previously proved that preosteoclast-released PDGF-BB could stimulate type H vessel formation, and drugs increasing preosteoclasts could promote bone formation in estrogen-deficient mice (Huang et al., 2018; Xie et al., 2014; Yin et al., 2018). In another study, a drug that promotes type H vessel formation in subchondral bone has been proven to attenuate osteoarthritis progression (Cui et al., 2016). In this study, we first observed the existence of type H vessels in callus and found that *A. muciniphila* stimulated the generation of this vessel subtype, which may be associated with the anti-inflammatory effect of *A. muciniphila* and why there was improved fracture repair with *A. muciniphila* treatment. We also found that the increase of angiogenesis induced by *A. muciniphila* was mainly attributed to the formation of type H vessels, which highlighted the importance of type H vessels in fracture healing. PDGF-BB, which has been proven to benefit vessel formation and fracture healing (Walia et al., 2018), was also detected in callus of *A. muciniphila*-treated mice. These data suggest that the increased number of PDGF-BB^+^ preosteoclasts induced by *A. muciniphila* might stimulate the formation of CD31^hi^Emcn^hi^ vessels in callus. Considering the involvement of type H vessels in bone healing, drugs that improve this specific capillary subtype or inhibit osteoclastogenesis (Huang et al., 2018; Yin et al., 2018) have the potential to improve fracture.

There are some limitations in our study. The difference in *A. muciniphila* abundance at 1 WPF between *A. muciniphila*-treated mice and vehicle-treated mice was not significant enough, whereas a significant increase in *A. muciniphila* abundance in mice feces appeared at 2 WPF. We hypothesized that many of the exogenously administered *A. muciniphila* may be eliminated from the mice body during the washout period (van der Lugt et al., 2019), and two occurrences of colonization may not be sufficient to provide enough *A. muciniphila* that can survive and grow in the host gut. On the other hand, it may also be possible that the transplantation of *A. muciniphila* alters the endogenous microbiota and preferentially enables the endogenous *A. muciniphila* to outcompete other bacteria, thus increasing the abundance of *A. muciniphila* in the mice gut after 2 weeks. Besides, the role of PDGF-BB^+^ preosteoclasts in *A. muciniphila*-boosted type H vessel formation has not been determined. This warrants further studies.

To sum up, we show that *A. muciniphila* improves type H vessel formation and bone repair, which may be associated with its effects in tightening the intestinal barrier and decreasing systemic inflammation at the early stage of fracture healing. Our study suggests that the *A. muciniphila* can be applied as a therapeutic agent for promoting fracture healing.

## MATERIALS AND METHODS

### Culture and characterization of *A. muciniphila*

We cultured *A. muciniphila* (ATCC, BAA-835) anaerobically at 37°C with constant shaking (135 rpm) in brain-heart-infusion broth containing 0.05% L-cysteine-HCl (Sigma-Aldrich) and 0.5% porcine mucin (Sigma-Aldrich), in accordance with the manufacturer's instructions and previous reports (Li et al., 2016a; Shen et al., 2017). The concentration of *A. muciniphila* was assessed by measuring the absorbance at the 600 nm wavelength (Li et al., 2016a).

To characterize the *A. muciniphila* mentioned above, centrifugation (8000 ***g***, 10 min, 4°C) was performed to collect a bacterial pellet. Total genomic DNA was obtained with a TIANamp Bacteria DNA Kit (Tiangen, DP302). PCR amplification product of *A. muciniphila* DNA was subjected to agarose gel electrophoresis and then a ChemiDoc XRS+ system (Bio-Rad) was used for DNA band imaging.

### Animals and treatments

Animal care and experimental procedures were approved by the Ethical Review Board at Xiangya Hospital, Central South University. All the animal experiments were conducted in the Department of Laboratory Animals, Central South University. C57BL/6 female mice (8-week-old) were used for surgical fracture and implantation, as described previously (Yuasa et al., 2015; Zhang et al., 2016). After general anesthesia by sodium pentobarbital intraperitoneal injection (50 mg/kg), an incision from the mid femur to the knee was made in the lateral right hindlimb of the mouse, and blunt dissection of muscle was performed to expose the mid-shaft of the femur. A sterilized 23-gauge (∼0.64 mm outer diameter) needle was drilled into the intercondylar notch of the distal femur. In bone marrow cavity, a needle was inserted along the axis of the femoral shaft until it penetrated through the proximal femur. A transverse fracture was made in the mid-shaft of the femur using sharp scissors. After we confirmed that the fracture was complete, the wound was sutured. For the *A. muciniphila* and *L. gasseri* (ATCC, 33323) groups, each mouse was orally administrated with 8×10^8^ colony-forming units (CFU) of *A. muciniphila* or *L. gasseri* in 0.5 ml PBS. An equivalent volume of PBS was given to each mouse in the vehicle group. These treatments were conducted twice a week. Mice were sacrificed under general anesthesia at 1, 2, 4 and 6 WPF. Whole-blood samples were collected by eyeball enucleation, and centrifugation (1000 ***g***, 4°C, 15 min) was performed to obtain serum samples, which were stored at −80°C before analysis.

For ABX treatment, mice were divided into two groups: ABX plus vehicle and ABX plus *A. muciniphila*. All mice were given a cocktail of broad-spectrum antibiotics with vancomycin (0.5 g/l, Macklin), ampicillin (1 g/l, Macklin), neomycin (1 g/l, Macklin) and metronidazole (1 g/l, Macklin) in drinking water for 2 weeks, followed by 2 days of washout period (Blacher et al., 2019). Fecal samples were placed on yeast extract, casitone and fatty acid (1 g casitone, 0.25 g yeast extract, 0.4 g NaHCO_3_, 0.1 g cysteine, 0.045 g K_2_HPO_4_, 0.045 g KH_2_PO_4_, 0.09 g NaCl, 0.009 g MgSO_4_**·**7H_2_O, 0.009 g CaCl_2_, 0.1 mg resazurin, 1 mg haemin, 1 μg biotin, 1 μg cobalamin, 3 μg p-aminobenzoic acid, 5 μg folic acid, 15 μg pyridoxamine, 0.2 g glucose, 0.2 g maltose and 0.2 g cellobiose, per 100 ml) agar plates (Browne et al., 2016) with 96 h anaerobic culturing to ensure fecal microbiota depletion. Then fracture surgery was conducted on all of the mice. As previous studies (Derrien et al., 2017; Geerlings et al., 2018) showed that *A. muciniphila* was found to resist vancomycin and metronidazole, a combination of vancomycin (0.5 g/l) and metronidazole (1 g/l) was given to all the mice until the end of the experiment to prevent the recolonization of environmental bacteria. For ABX plus *A. muciniphila* group mice, *A. muciniphila* treatment started on the day of surgery. *A. muciniphila* (8×10^8^ CFUs in 0.5 ml PBS) was given by oral gavage to each mouse in the ABX plus *A. muciniphila* group, and the same volume of PBS was given to mice in the ABX plus vehicle group, twice a week, for a total of 6 weeks.

In the DSS experiment, all the mice were divided into three groups: vehicle group, DSS plus vehicle group and DSS plus *A. muciniphila* group. For mice in the DSS plus vehicle and DSS plus *A. muciniphila* groups, 2.5% (w/v) DSS (40 kDa, Sigma-Aldrich, 52423) was added into drinking water *ad libitum*. A 7-day DSS treatment was given to the DSS plus vehicle and DSS plus *A. muciniphila* groups before fracture operation. After surgery, three cycles of treatment (8-day normal drinking water followed by 6-day DSS) were performed until the experimental end point (Chen et al., 2020; Liu et al., 2019). Mice in the vehicle group received normal drinking water during the whole experiment. *A. muciniphila* (8×10^8^ CFUs) in 0.5 ml PBS was orally administered to each mouse in the DSS plus *A. muciniphila* group twice a week. An equivalent volume of PBS was given to each mouse in the vehicle and DSS plus vehicle groups. All the mice were weighed twice a week until sacrificed.

### Quantification of *A. muciniphila* in feces and 16S rDNA qRT-PCR analysis

Fecal genomic DNA was extracted with a TIANamp stool DNA kit (Tiangen, DP328). qRT-PCR was carried out to amplify the specific *A. muciniphila* gene sequence with a pair of specific primers (forward 5′-CCTTGCGGTTGGCTTCAGAT-3′ and reverse 3′-CAGCACGTGAAGGTGGGGAC-5′) (Grander et al., 2018) using FastStart Universal SYBR Premix ExTaq (Takara Biotechnology) (Chen et al., 2018; Hu et al., 2018). The cycle threshold (CT) value of each sample was then compared with a standard curve made by diluting genomic DNA of *A. muciniphila*. Log_10_ of *A. muciniphila* number per gram of fecal content was used to indicate the abundance of *A. muciniphila* (Grander et al., 2018; Li et al., 2016a).

qRT-PCR analysis with 16S rDNA was performed as described previously (Bacchetti De Gregoris et al., 2011). Briefly, a fecal DNA sample was diluted 1:4. These two samples (undiluted sample and diluted sample) were subjected to qRT-PCR for testing the amplification efficiency of each primer pair (Fig. S2) with the following formulas:
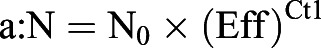

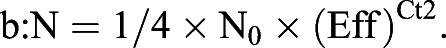
Here, N is a constant that refers to the threshold of qRT-PCR and N_0_ refers to the original DNA content of the template. Eff represents amplification efficiency. Ct1 and Ct2 represent the cycle threshold (Ct) value of undiluted sample and diluted sample, respectively. Eff of each primer pair can be calculated using the formula below (formula a/formula b):
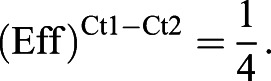
The percentage of each taxonomic group in feces (X) was calculated by Ct value and amplification efficiency of each primer pair with the formula:
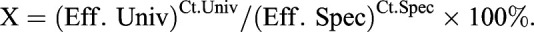
Eff. Univ and Eff. Spec represent the amplification efficiency of universal primers and taxon-specific primers. Ct. Univ and Ct. Spec represent the Ct value of universal primers and taxon-specific primers. Primers used for 16S rDNA qRT-PCR are listed in Table S1.

### Micro-computed tomography (µCT) analyses

Fractured femora harvested from mice were fixed overnight in 4% paraformaldehyde and analyzed by µCT (SCANCO Medical AG, vivaCT 80) as described in our previous studies (Huang et al., 2018; Rao et al., 2018). The scanner was set at 55 kV, a current of 145 µA and voxelsize of 11.4 µm, respectively. A total of 600 slices were analyzed and the fracture line was set in the middle of this range. The region of interest was selected slice-by-slice and a fixed threshold (60) was used to define mineralized tissue in callus. TV, BV and BV/TV of callus were calculated.

### Biomechanical test

After µCT scanning, a four-point bending test was performed to test the fracture-healing quality of femora using a computer-controlled mechanical testing machine (Instron, 3343M1372) (Zhang et al., 2016). In brief, femora were placed in the anterior-posterior direction (patella side facing up) 9 mm apart on the lower supporting bars. The span of the upper bars measured 5.5 mm. A constant vertical compression load was applied at a speed of 3 mm/min until failure occurred. The values of maximum bending load (N) were recorded automatically by computer.

### Histological, immunohistochemical and immunofluorescent analyses

For the histological and immunohistochemical staining, dissected femora were fixed in 4% paraformaldehyde overnight and decalcified in 0.5 M EDTA (pH 7.4) at 4°C, with constant shaking for ∼3 days. After that, dehydration was performed using a graded series of ethanols, followed by paraffin embedding. Bone samples were sliced (5 μm) longitudinally and processed for TRAP staining (Sigma-Aldrich, 387A) to measure osteoclast formation in accordance with our established protocols (Huang et al., 2018; Rao et al., 2018). TRAP^+^ mononuclear cells were defined as preosteoclasts, and TRAP^+^ multinucleated cells (at least three nuclei) were identified as osteoclasts.

The level of inflammatory responses in bone tissues was tested by IL1β, IL6 and TNF-α immunohistochemical analyses as reported previously (Rao et al., 2018). Antibodies against IL1β (16806-1-AP, 1:50), IL6 (21865-1-AP, 1:200) and TNF-α (17590-1-AP, 1:100) were obtained from ProteinTech, and the secondary antibody was goat polyclonal secondary antibody against rabbit IgG H&L (Alexa Fluor 594), pre-adsorbed (Abcam, ab150088, 1:200).

For Safranin O staining (Yuasa et al., 2015), deparaffinization and rehydration were performed using a standard protocol. Sections were placed in hematoxylin for 2 min and washed in running tap water for 5 min. After 0.1% Safranin O (Sigma-Aldrich, S2255) staining for 3 min, and 1% acetic acid rinsing for 10-15 s, sections were stained with 0.1% Fast Green solution (Sigma-Aldrich, F7258) for 5 min, followed by washing in running tap water for 5 min. Finally, dehydrated sections were coverslipped with Permount and images were acquired using an Olympus CX31 optical microscope.

For analysis of colonic damage induced by DSS, colon samples were fixed in 4% paraformaldehyde at 4°C overnight. After rinsing in PBS three times, dehydration and paraffin embedding were performed as described above. Colon samples were transversely sectioned (5 μm) and processed for HE staining. Scoring was conducted as described previously (Welz et al., 2011). In brief, we added scores of inflammation and tissue damage on different levels from 0 to 3, from which the total colon histological scores of 0 to 6 were obtained. Inflammation scores were defined as: 0, no inflammatory infiltration detected in the lamina propria; 1, presence of inflammatory cells in the mucosa; 2, inflammatory infiltration in the submucosa; and 3, transmural inflammatory infiltration. Tissue damage scores were defined as: 0, no mucosal damage; 1, discrete lesions in the colonic epithelium; 2, extended epithelial damage detected in elongated crypt-containing areas, crypt abscesses or focal ulceration; and 3, extended ulceration in the colonic wall. Image-Pro Plus software (version 6.0) was employed to measure staining intensity and positive-stained cells or area in quantitative analyses of histological and immunohistochemical staining.

For all immunofluorescent staining, femora were fixed in 4% paraformaldehyde for 4 h and decalcified in 0.5 M EDTA (pH 7.4) at 4°C, with constant shaking for 2 to 3 days. Bone samples were immersed in 30% sucrose overnight for dehydration. After exposure to liquid nitrogen for a few seconds, samples were embedded in optimal cutting temperature compound (Sakura Finetek) and sectioned at the corresponding thickness of each assay. Sections (5 μm) were incubated with OCN antibody (1:200, Abcam, ab93876) overnight at 4°C. Then, the secondary antibody Cy3 AffiniPure Goat Anti-Rabbit IgG (H+L) (Jackson ImmunoResearch, 111-165-003, 1:300) was incubated at room temperature for 1 h while protected from light. Slices (10 μm) were stained with TRAP antibody (Santa Cruz, sc-30833, 1:200) and PDGF-BB antibody (Abcam, ab21234, 1:50), as well as the corresponding secondary antibodies donkey anti-Goat IgG H&L (Alexa Fluor 488) (Abcam, ab150129, 1:300) and Cy3 AffiniPure goat anti-Rabbit IgG (H+L) (Jackson ImmunoResearch, 111-165-003, 1:300). For type H vessel immunofluorescent staining (Ramasamy et al., 2014), 30 μm sections were cut. Primary antibodies against CD31 (Abcam, ab28364, 1:50) and Emcn (Santa Cruz, V.7C7, 1:100), Cy3 AffiniPure Goat anti-rabbit IgG (H+L) (Jackson ImmunoResearch, 111-165-003, 1:300) and Alexa Fluor 488 AffiniPure donkey anti-rat IgG (H+L) (Jackson ImmunoResearch, 712-545-150, 1:300) were applied. Sections were observed using a Zeiss Axio Imager 2 fluorescence microscope. The total vessel area (TVA; positive for Emcn), callus area (CA), type H vessel area (HVA; positive for Emcn and CD31) and non-type H vessel area (NHA; positive for Emcn and negative for CD31) were measured using Image-Pro Plus software (version 6.0) and Image J software (version 1.51) to calculate TVA/CA, HVA/TVA and NHA/CA (Huang et al., 2018). The numbers of positive stained cells and relative staining intensity were determined in three sections per mouse and three mice per group.

### qRT-PCR analyses

Colonic RNA was extracted using TRIzol Reagent (Invitrogen), and a RevertAid First Strand cDNA Synthesis kit (Fermentas) was used to synthesize cDNA. Next, FastStart Universal SYBR Premix ExTaqTM II (Takara Biotechnology) with an ABI PRISM 7900HT System (Applied Biosystems) was employed to conduct qRT-PCR. Relative gene expression was measured using the 2^–ΔΔCT^ method with GAPDH used as a control. qRT-PCR primers used in this study were as follows (Li et al., 2016b; Morhardt et al., 2019; Plovier et al., 2017): for *Cldn2*, 5′-TCTCAGCCCTGTTTTCTTTGG-3′ (forward) and 5′-GGCGAGCAGGAAAAGCAA-3′ (reverse); for *Cldn3*, 5′-TCATCACGGCGCAGATCA-3′ (forward) and 5′-CTCTGCACCACGCAGTTCA-3′ (reverse); for *Cldn15*, 5′-GGCGGCATCTGTGTCTTCTC-3′ (forward) and 5′-TGGTGGCTGGTTCCTCCTT-3′ (reverse); for *Ocln*, 5′-ATGTCCGGCCGATGCTCTC-3′ (forward) and 5′-TTTGGCTGCTCTTGGGTCTGTAG-3′ (reverse); for *Jam3*, 5′-CACTACAGCTGGTACCGCAATG-3′ (forward) and 5′-CTGGGATTGGCTCTGGAATC-3′ (reverse); for *Rankl*, 5′-CCTGATGAAAGGAGGGAGCA-3′ (forward) and 5′-TGGAATTCAGAATTGCCCGA-3′ (reverse); for *Tnfα*, 5′-AACTCCAGGCGGTGCCTAT-3′ (forward) and 5′-TGCCACAAGCAGGAATGAGA-3′ (reverse); for *Il-10*, 5′-CCCTTTGCTATGGTGTCCTT-3′ (forward) and 5′-TGGTTTCTCTTCCCAAGACC-3′ (reverse); for *Il17a*, 5′-TGACGCCCACCTACAACATC-3′ (forward) and 5′-CATCATGCAGTTCCGTCAGC-3′ (reverse); and for *Gapdh*, 5′-CACCATGGAGAAGGCCGGGG-3′ (forward) and 5′-GACGGACACATTGGGGGTAG-3′ (reverse).

### Gut permeability assays

We used FITC-dextran (Sigma-Aldrich, FD4) to assess barrier function by oral administration as described previously (Li et al., 2016b). Briefly, each mouse was given 0.5 ml of 25 mg/ml FITC-dextran, and serum samples were collected 4 h later under general anesthesia. The serum dextran level was determined by comparison with the standard curve, which was developed by serial dilutions of known concentration of FITC-dextran using a spectrophotometer (Varioskan LUX, Thermo Scientific) with excitation at 490 nm and emission at 520 nm. To quantify the LPS level in the serum of mice, a LAL assay kit purchased from GeneScript was used in accordance with the manufacturer's instruction.

### ELISA

The concentrations of serum TNF-α, IL10 and M-CSF were evaluated by using commercial ELISA kits (Multisciences Biotech). Serum RANKL, OCN and CTX-1 levels were tested with ELISA kits purchased from Elabscience. Another ELISA kit (Cusabio) was applied to measure the level of SAA3. All the procedures were conducted according to the manufacturer's instructions.

### Statistical analyses

All data were presented as mean±s.d. An unpaired two-tailed Student's *t*-test was used to analyze the differences between two groups. One-way or two-way ANOVA with Bonferroni post-hoc test was used for multiple-group comparisons. For all experiments, *P*<0.05 was considered to be significant. GraphPad Prism software (version 6.01) was used for statistical analyses.

## Supplementary Material

Supplementary information
