# Supplementary material for: Akkermansia muciniphila promotes type H vessel formation and bone fracture healing by reducing gut permeability and inflammation
Source: Dis Model Mech. 2020 Dec 1;13(11):dmm043620. doi: 10.1242/dmm.043620 (PMC7725610; doi:10.1242/dmm.043620)
Supplement: Supplementary information [file dmm-13-043620-s1.pdf]

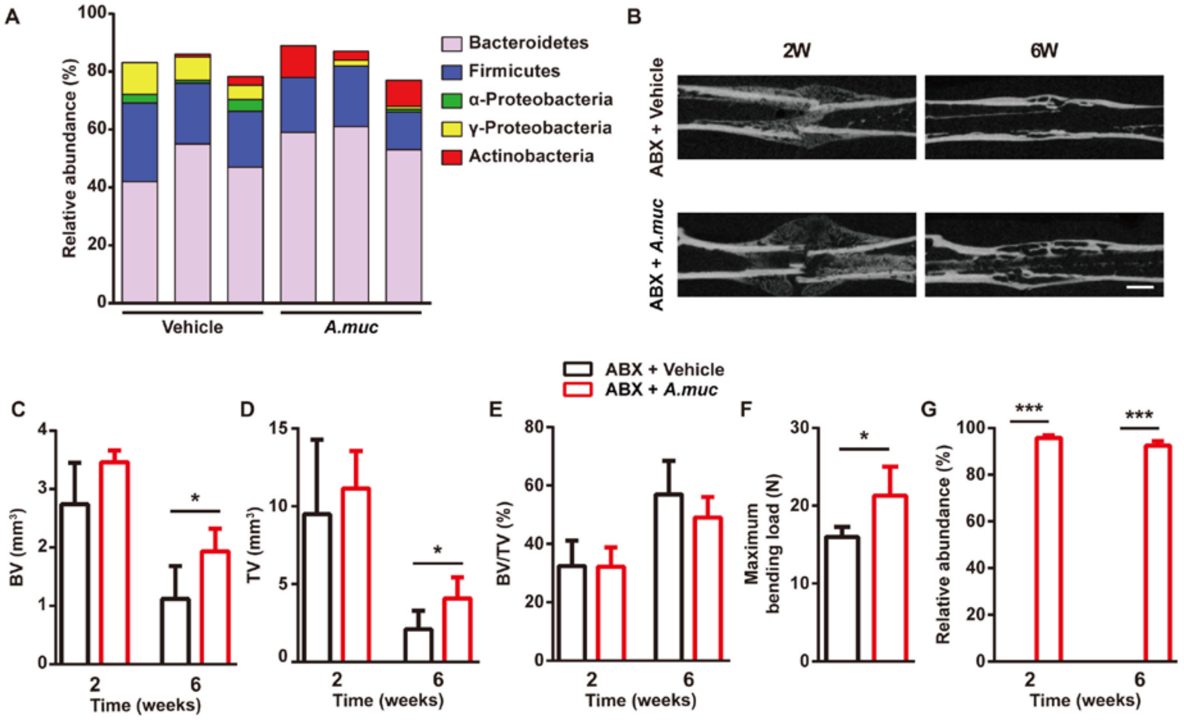

Fig. S1. ***A. muc*** **improves bone repair in gut microbiota-depleted mice.** (A) Fecal microbiota composition of vehicle-treated group and *A. muc*-treated group evaluated by 16S rDNA qRT-PCR. *n* = 3 per group. (B) Representative µCT images of fractured femora from ABX + vehicle and ABX + *A. muc* group mice at 2 and 6 weeks. Scale bar: 1 mm. (C-E) µCT quantitative analyses of BV (C), TV (D) and BV/TV (E) of fractured femora at 2 and 6 WPF. *n* = 5 per group. (F) Four-point bending measurement of femoral ultimate load at 6 WPF. *n* = 5 per group. (G) Abundances of *A. muc* in fecal samples of ABX + vehicle and ABX + *A. muc* group mice. *n* = 5 per group. $^{*}P < 0.05$, $^{***}P < 0.001$ by unpaired two tailed Student's *t* test.

Disease Models & Mechanisms • Supplementary information

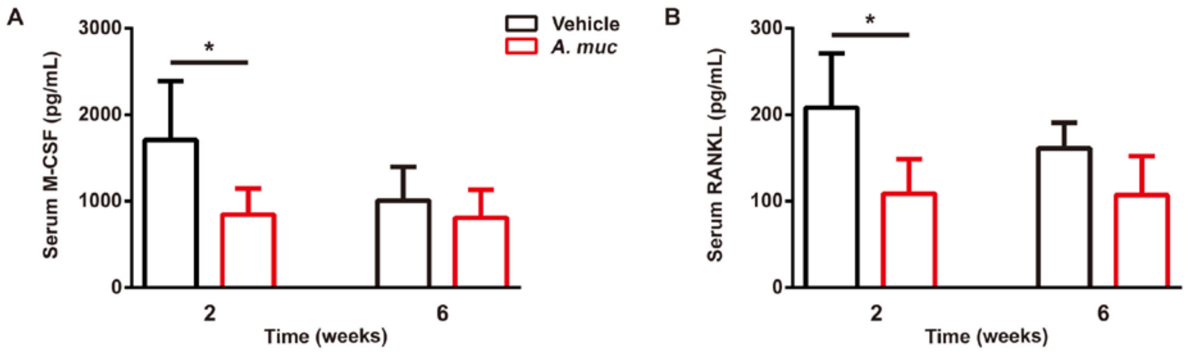

Fig. S2. *A. muc* decreases circulatory levels of M-CSF and RANKL. (A) Serum concentrations of M-CSF (A) and RANKL (B) detected by ELISA at 2 and 6 WPF. $n = 5$ per group. $^{*}P < 0.05$ by unpaired two tailed Student's t test.

Disease Models & Mechanisms • Supplementary information

Table S1: Primers used for 16S rDNA qRT-PCR in Figure S1

Target group	Primer sequence
Universal	AAACTCAAAKGAATTGACGG<br>CTCACRRCACGAGCTGAC
α-Proteobacteria	CIAGTGTAGAGGTGAAATT<br>CCCCGTCAATTCCTTTGAGTT
γ-Proteobacteria	TCGTCAGCTCGTGTYGTGA<br>CGTAAGGGCCATGATG
Bacteroidetes	CRAACAGGATTAGATACCCT<br>GGTAAGGTTCCTCGCGTAT
Firmicutes	TGAAACTYAAAGGAATTGACG<br>ACCATGCACCACCTGTC
Actinobacteria	TACGGCCGCAAGGCTA<br>TCRTCCCCACCTTCCTCCG
